# Activated mesenchymal stem/stromal cells promote myeloid cell differentiation via CCL2/CCR2 signaling

**DOI:** 10.1016/j.stemcr.2024.02.002

**Published:** 2024-02-29

**Authors:** Satoshi Yamazaki, Yo Mabuchi, Takaharu Kimura, Eriko Grace Suto, Daisuke Hisamatsu, Yuna Naraoka, Ayako Kondo, Yuzuki Azuma, Riko Kikuchi, Hidekazu Nishikii, Soji Morishita, Marito Araki, Norio Komatsu, Chihiro Akazawa

**Affiliations:** 1 Laboratory of Stem Cell Therapy, Faculty of Medicine, University of Tsukuba, Ibaraki 305-8575, Japan; 2 Intractable Disease Research Center, Juntendo University Graduate School of Medicine, Hongo, Bunkyo-ku, Tokyo 113-8421, Japan; 3 Department of Clinical Regenerative Medicine, Fujita Medical Innovation Center, Fujita Health University, Tokyo 144-0041, Japan; 4 Department of Hematology, Institute of Medicine, University of Tsukuba, Ibaraki 305-8575, Japan; 5 Laboratory for the Development of Therapies against MPN, Juntendo University Graduate School of Medicine, Hongo, Bunkyo-ku, Tokyo 113-8421, Japan; 6 Department of Advanced Hematology, Juntendo University Graduate School of Medicine, Hongo, Bunkyo-ku, Tokyo 113-8421, Japan

## Abstract

Myeloid cells, which originate from hematopoietic stem/progenitor cells (HSPCs), play a crucial role in mitigating infections. This study aimed to explore the impact of mesenchymal stem/stromal cells (MSCs) on the differentiation of HSPCs and progenitors through the C-C motif chemokine CCL2/CCR2 signaling pathway. Murine MSCs, identified as PDGFRα^+^Sca-1^+^ cells (PαS cells), were found to secrete CCL2, particularly in response to lipopolysaccharide stimulation. MSC-secreted CCL2 promoted the differentiation of granulocyte/macrophage progenitors into the myeloid lineage. MSC-derived CCL2 plays an important role in the early phase of myeloid cell differentiation *in vivo*. Single-cell RNA sequencing analysis confirmed that CCL2-mediated cell fate determination was also observed in human bone marrow cells. These findings provide valuable insights for investigating the *in vivo* effects of MSC transplantation.

## Highlights

• Activated mouse MSCs stimulate HSPC differentiation
• Lipopolysaccharide-treated MSCs enhanced CCL2 secretion by mouse MSCs
• MSC-derived CCL2 promotes GMP engraftment and differentiation *in vivo*
• CCL2-mediated cell fate changes are induced in human HSPCs

## Graphical abstract

**Figure:**
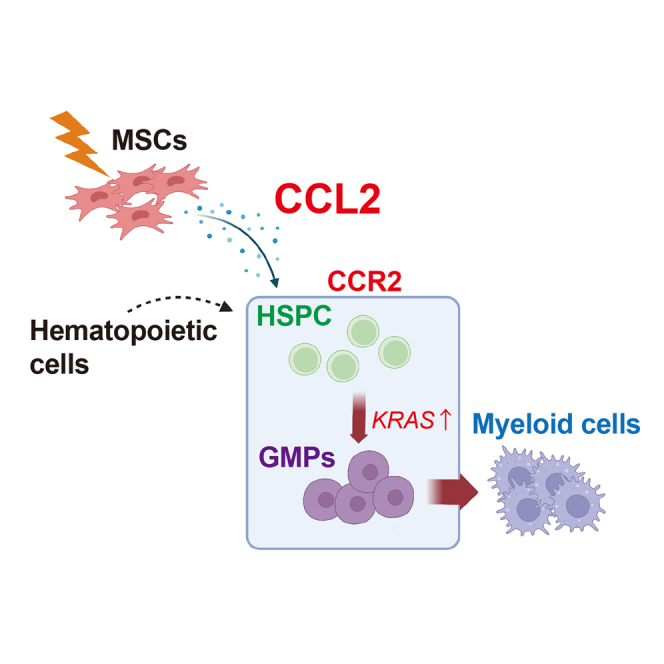

## Teaser

In this article, Akazawa and colleagues show that murine mesenchymal stem/stromal cells (MSCs), identified as PDGFRα^+^Sca-1^+^ cells (PαS cells), secrete the chemokine CCL2 in response to lipopolysaccharide stimulation. CCL2 promotes the differentiation of granulocyte/macrophage progenitors into the myeloid lineage, playing a crucial role in the early phase of myeloid cell differentiation *in vivo*. Single-cell RNA sequence analysis in human bone marrow cells confirms the significance of MSC-derived CCL2 in cell fate determination, providing valuable insights on the *in vivo* effects of MSC transplantation.

## Introduction

Hematopoietic stem/progenitor cells (HSPCs) maintain the homeostasis of the hematopoietic system in the bone marrow (BM). The ability of HSPCs to engraft and sustain long-term hematopoiesis is the basis of BM transplantation (Osawa et al., 1996; Wilkinson et al., 2019). BM transplantation therapy promotes platelet generation and early neutrophil differentiation, thereby reducing the risk of infection (Zimmerli et al., 1991). Myeloid differentiation can also be induced through the administration of specific factors, such as granulocyte colony-stimulating factor (G-CSF) and granulocyte/macrophage colony-stimulating factor (GM-CSF), at the time of HSPC transplantation (Mehta et al., 2015). However, it remains unclear which factors contribute to lineage determination in BM or transplanted cells.

Mesenchymal stem/stromal cells (MSCs) are tissue stem cells present in multiple regions of the body, including the BM (Mabuchi and Matsuzaki, 2016; Mabuchi et al., 2013; Pittenger et al., 1999). Stromal cells, including MSCs, directly interact with HSPCs and maintain their undifferentiated state *in vivo* (Mendez-Ferrer et al., 2010; Omatsu et al., 2014; Sugiyama et al., 2006; Zhou et al., 2014). MSCs physically support the differentiation and maturation of HSCs through cytokines, exosomes, and extracellular matrix proteins (Teleb et al., 2023). Previous studies have demonstrated that MSCs promote the proliferation of HSCs *in vitro* (da Silva et al., 2005). Recent reports have shown that C-C motif chemokine ligand 2 (CCL2)-producing stromal cells in the BM express toll-like receptors and are involved in monocyte migration from the BM (Shi et al., 2011). MSCs are attracting attention as a cell source for transplantation therapy, which is already being used to treat many diseases (Bianco et al., 2008). However, there is a paucity of knowledge regarding the characteristics of these cells and how they affect surrounding cells.

This study aimed to unravel the mechanisms underlying the effects of MSCs on HSPC differentiation. We found that activated PDGFRα^+^Sca-1^+^ (PαS) cells secreted CCL2 and regulated the differentiation ability of HSPCs to produce myeloid cells. When CCL2 was knocked out of PαS cells, the ability of HSPCs to differentiate into myeloid cells was significantly reduced. The main cell cluster secreting CCL2 stimulated by lipopolysaccharide (LPS) was PαS cells, which regulate induction of differentiation by targeting granulocyte/macrophage progenitors (GMPs). Further, the MSCs acted as sensors against inflammation and infection and propagated inflammatory signals to hematopoietic cells *in vivo*. This supports a model in which human MSCs, through CCL2 signaling, can promote myeloid cell differentiation and support the recovery of essential immune cell compartments.

## Results

### Factors secreted by MSCs promote myeloid cell differentiation

**Figure 1:**
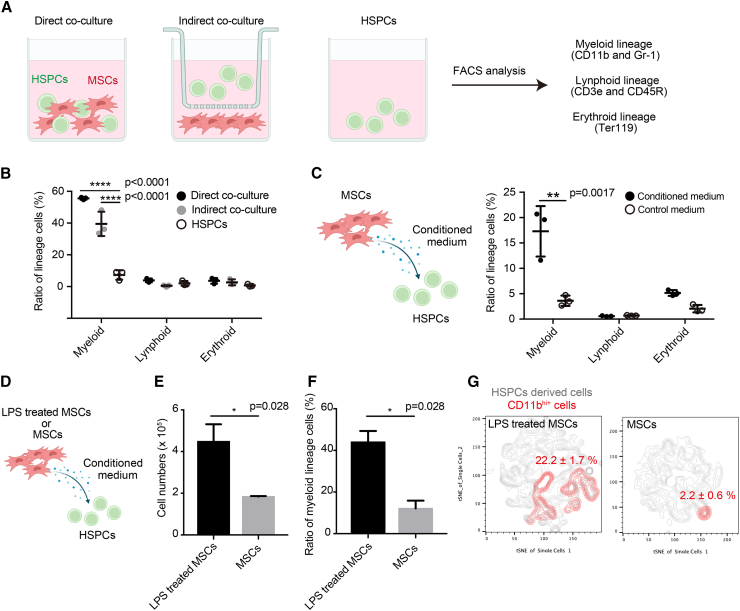
Secretory factors released from mesenchymal stem cells (MSCs) promote the differentiation of hematopoietic stem/progenitor cells (HSPCs) into myelocyte lineage (A) Experimental scheme of the analysis of cultured cells *in vitro* for culturing HSPCs. HSPCs (c-Kit^+^Sca-1^+^Lineage^-^cells: KSL cells) were isolated from adult enhanced green fluorescent protein (EGFP) mouse bone marrow (BM). HSPCs were cultured directly on MSCs (CD45^-^CD31^-^Ter119^-^PDGFRα^+^Sca-1^+^ cells: PαS) (direct co-culture) or indirectly on PαS cells using a cell culture insert dish (indirect co-culture). HSPCs were uniquely cultured as control (HSPCs). (B) The graph shows the proportion of hematopoietic lineage cells (black: direct co-culture, gray: indirect co-culture, and white: HSPCs alone). Data are representative of three independent experiments (n = 3). (C) *In vitro* culture assay of HSPCs supplemented with the culture supernatant of MSCs. Control medium is a normal hematopoietic culture medium. The graph shows the proportion of hematopoietic lineage cells (black: conditioned medium, and white: control medium). Data are representative of three independent experiments (n = 3). (D) Analysis of HSPCs in culture with conditioned medium of lipopolysaccharide (LPS)-stimulated MSCs (LPS administration, 100 ng/mL). (E and F) The graph shows the cell number (E) and proportion of myeloid lineage cells (F). Data are representative of three independent experiments (n = 5). (G) Cell surface expression changes in cultured HSPCs after adding conditioned medium. The cluster analysis of cell surface antigen was performed using FACS t-SNE (FlowJo software [v10.5.3]). Data are shown as mean ± standard error of mean (SEM). See also Figure S1.

HSPCs are present in an undifferentiated state in the BM niche (Schofield, 1978). Within this niche, MSCs can control HSPC differentiation (Omatsu et al., 2014). To elucidate the mechanism behind this interaction, HSPCs (c-Kit^+^Sca-1^+^Lineage^-^cells: KSL cells) and MSCs (CD45^-^CD31^-^Ter119^-^PDGFRα^+^Sca-1^+^ cells: PαS cells) were isolated using flow cytometry and co-cultured *in vitro* (Figure 1A). In direct and indirect co-culture with PαS cells, KSL cells differentiated into the myeloid lineage (CD11b^+^Gr-1^+^ cells), whereas KSL cells cultured alone did not differentiate (Figure 1B). In contrast, the ratio of differentiation into the lymphoid lineage (CD3e^+^CD45R^+^ cells) and erythroid lineage (Ter119^+^ cells) did not change (Figure 1B). KSL cells were incubated with conditioned media isolated from cultured PαS cells to investigate the effect of secreted factors of MSCs on HSPC differentiation, and the MSC-conditioned media induced the differentiation of HSPC cells into myeloid lineage cells (Figure 1C).

To investigate the factors secreted by MSCs, we performed analysis using culture supernatants obtained from PαS cells following stimulation with LPS. Addition of the culture supernatant of LPS-treated MSCs to HSPCs increased the number of cells and percentage of myeloid lineage cells compared with those in non-treated MSCs (Figure 1D). Particularly, the CD11b-positive cell cluster significantly increased the population (Figures 1E–1G and S1A–S1D). These results showed that MSC-secreted factors promoted the differentiation of HSPCs into myeloid cells.

### Myeloid differentiation is regulated by CCL2 secreted from MSCs

**Figure 2:**
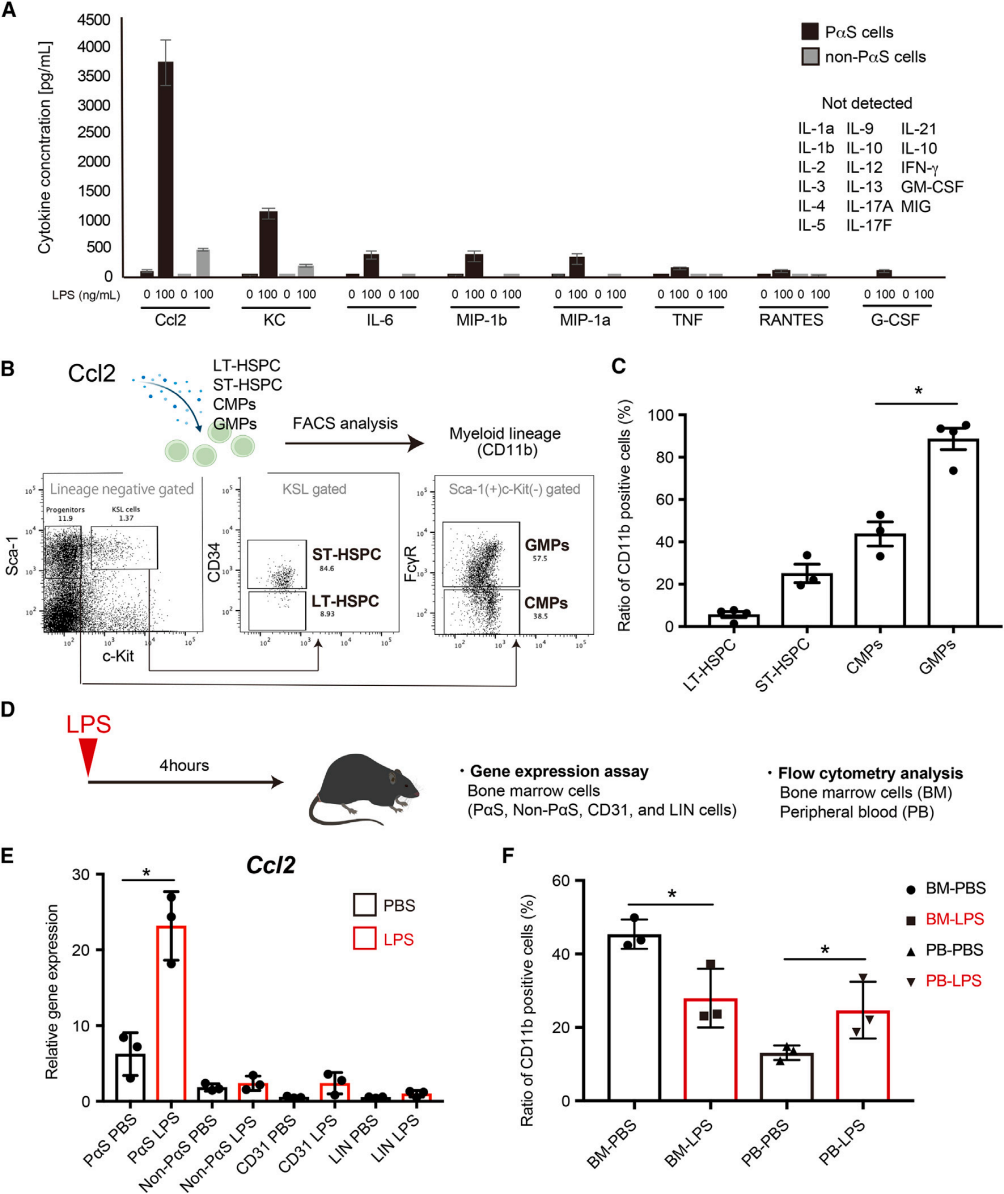
PDGFRα^+^Sca-1^+^ (PαS) cells secrete CCL2 after lipopolysaccharide (LPS) administration (A) Cytokines secreted from PαS and non-PαS cells following LPS administration (0 ng/mL and 100 ng/mL) were measured using a cell-based assay. The graph shows the concentration of detected cytokines (pg/mL) (n = 3). (B) Mouse mesenchymal stem cell (MSC) culture supernatant promoted myeloid differentiation. FACS profile of hematopoietic progenitor cells cultured with conditioned medium (mouse CCL2). (C) Bar graph summarizes the myeloid cell ratio in FACS profile (LT-hematopoietic stem/progenitor cell [HSPC], ST-HSPC, CMPs, and granulocyte/macrophage progenitors [GMPs]) (n = 3). (D) Experimental scheme of the analysis of the effect of LPS stimulation on bone marrow (BM) and peripheral blood (PB) cells. LPS was intraperitoneally injected, and BM and PB cells were harvested 4 h after injection. (E) *CCL2* gene expression levels in BM cells (PαS cells, non-PαS cells, CD31^+^ endothelial cells, lineage+ mature hematopoietic cells, and PI-living cells). RNA was extracted from each cell type and compared and analyzed using quantitative polymerase chain reaction (qPCR) (n = 3). (F) Bar graph of the myeloid cell ratio *in vivo* after LPS or phosphate-buffered saline (PBS) administration (BM and PB) (n = 3). Data are shown as mean ± standard error of mean (SEM). ^∗^p < 0.05. See also Figure S2.

We characterized the secreted factors induced upon treatment of PαS cells with 100 ng/mL LPS and compared them with those in the non-PαS cell fraction (cell population excluding PDGFRα^+^Sca-1^+^ cells in non-hematopoietic cells). The production of the cytokines CCL2, mouse keratinocyte-derived chemokine (KC), interleukin (IL)-6, macrophage inflammatory protein (Mip)-1a, Mip-1b, tumor necrosis factor, regulated on activation, normal T cell expressed and secreted (RANTES), and G-CSF was induced by LPS treatment. CCL2 production was particularly higher in PαS cells than in non-PαS cells (Figure 2A). Next, we examined hematopoietic cells that can affect the ability of myeloid differentiation. We added culture supernatants of 293T cells overexpressing mouse CCL2 (mCCL2) to hematopoietic cells. Mouse BM cells were separated into CD34-negative long-term HSPCs (LT-HSPC), CD34-positive short-term HSPCs (ST-HSPC), common myeloid progenitors (CMPs), and GMPs (Figure 2B). In the GMP population, mCCL2 promoted differentiation into CD11b-positive myeloid lineage (Figure 2C). Myeloid differentiation of HSPCs is similar to that of blood cells during inflammation (Iwasaki and Akashi, 2007). Therefore, we analyzed the effects of inflammation on blood and mesenchymal cells in the acute inflammation model (Figures 2D and S2A). The expression of *Ccl2* was analyzed to determine the contribution of CCL2 secretion in the BM population (PaS, non-PaS, CD31, and lineage-positive cells) (Figures 2E and S2B). Four hours after LPS administration, CD11b-positive cells did not express CCL2 in the BM. As a result, CCL2 expression was high in mouse MSCs (PαS compartment) (Figure 2E). The proportion of CD11b^+^ cells decreased in the BM but increased in peripheral blood (PB) cells from the LPS-treated group (Figure 2F). The proportions of MSCs and CD31^+^ cells in the BM did not change in the LPS-treated group compared to those in the non-LPS-treated group, whereas the percentage of HSPCs increased (Figures S2C and S2D). Immunohistochemical analysis was performed to determine the contribution of CCL2 secretion in BM cells. PDGFRα-positive cells secreted CCL2 in the BM (Figures S2E and S2F). These results suggest that the main source of CCL2 in the BM is PDGFRα-positive MSCs.

### CCR2-deficient BM cells inhibit GMP differentiation

The chemokine receptor, CCR2, mediates the inflammatory response of monocytes, and it is activated by CCL2 (Serbina and Pamer, 2006). The expression pattern of CCR2 receptor in the hematopoietic subpopulation contained in BM cells was confirmed using flow cytometry, showing that the CCR2 receptor was highly expressed in GMPs and monocytes (Figure S3A). Integrated gene analysis via Gene Expression Commons (Seita et al., 2012) confirmed the high expression of the *Ccr2* gene in GMPs and monocytes (Figure S3B).

**Figure 3:**
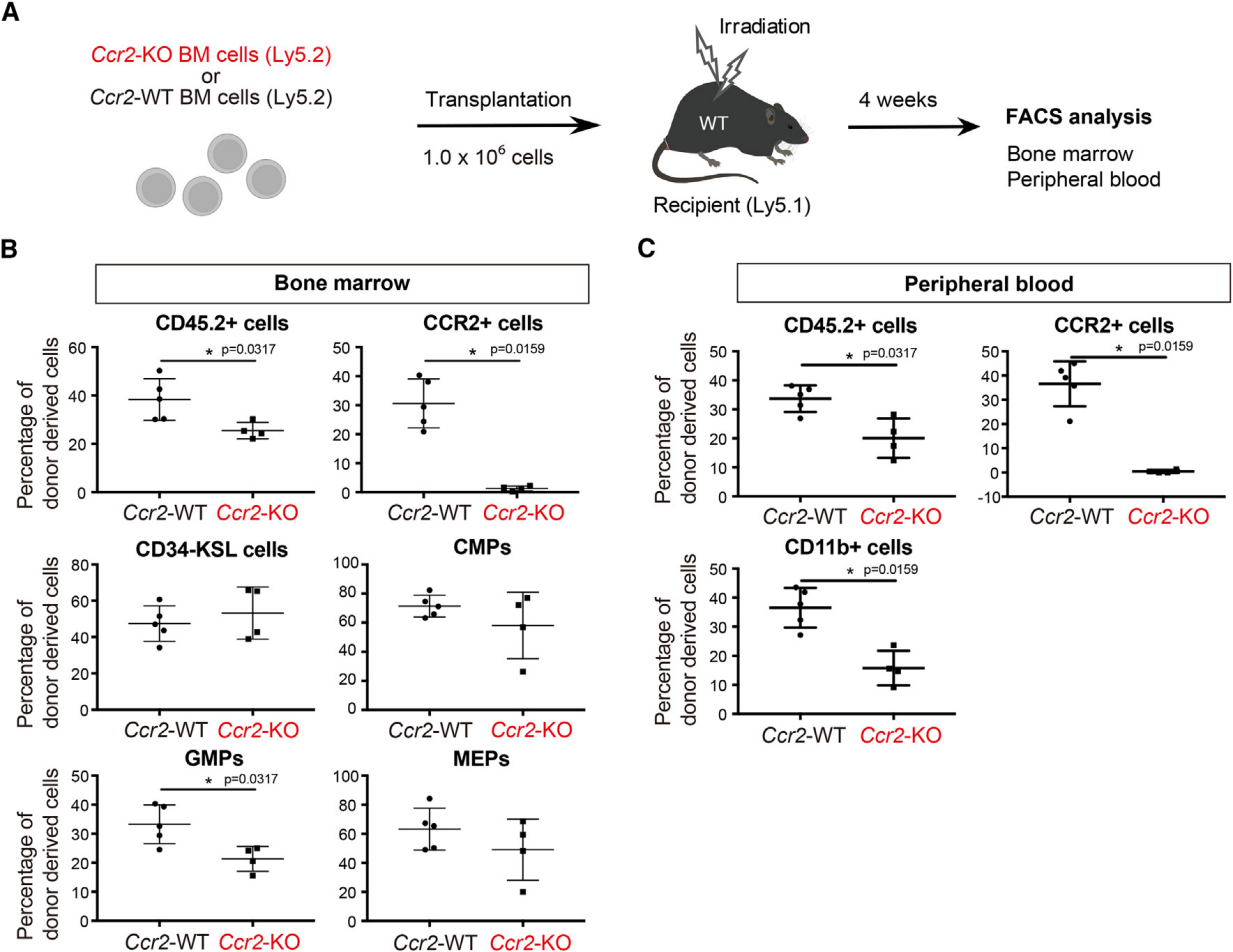
*Ccr2* deficiency inhibits lineage differentiation of myeloid cells after BM cell transplantation (A) Analysis of the differentiation potential of transplanted cells after BM cell transplantation. BM cells were collected from *Ccr2* knockout (*Ccr2*-KO) or wild-type (*Ccr2*-WT) mice (both Ly5.2) and transplanted into irradiated recipient WT mice (Ly5.1) together with BM cells from F1 mice (Ly5.1/Ly5.2). (B and C) Fractions of hematopoietic stem/progenitor cells (HSPCs) and progenitors among Ly5.2 and F1 BM cells in transplanted mice were analyzed 4 weeks after transplantation. Each dot shows the percentage of cells within the *Ccr2*-WT or *Ccr2*-KO Ly5.2^+^ donor-derived population (B: bone marrow, C: peripheral blood). Error bars represent standard error of mean (*Ccr2*-WT: n = 5 or *Ccr2*-KO: n = 4). ^∗^Unpaired Student’s t test. Data are shown as mean ± standard error of mean (SEM). ^∗^p < 0.05. See also Figure S3.

To elucidate the role of CCL2/CCR2 signaling in hematopoietic cells, we performed *in vivo* cell transplantation experiments using *Ccr2*-knockout cells. *Ccr2*-deficient mouse-derived BM cells (*Ccr2-*KO BM cells：Ly5.2) and wild-type (WT) mouse-derived BM cells (*Ccr2*-WT BM cells：Ly5.2) were separately transplanted into lethally irradiated mice (recipient WT mice：Ly5.1) (Figure 3A). After 4 weeks of transplantation, the proportion of transplanted *Ccr2-*KO BM cells was lower than that in *Ccr2*-WT BM cells (CD45.2 cells) (Figure 3B). The proportions of HSCs (CD34^-^KSL cells), CMPs, and MEP cells did not differ, predominantly due to a lower proportion of GMPs (Figure 3B). Flow cytometry of PB cells revealed that the ratio of CD11b^+^ macrophages decreased in *Ccr2-*KO BM transplanted mice (Figure 3C).

### Control of hematopoietic cell fate via MSC-derived CCL2

**Figure 4:**
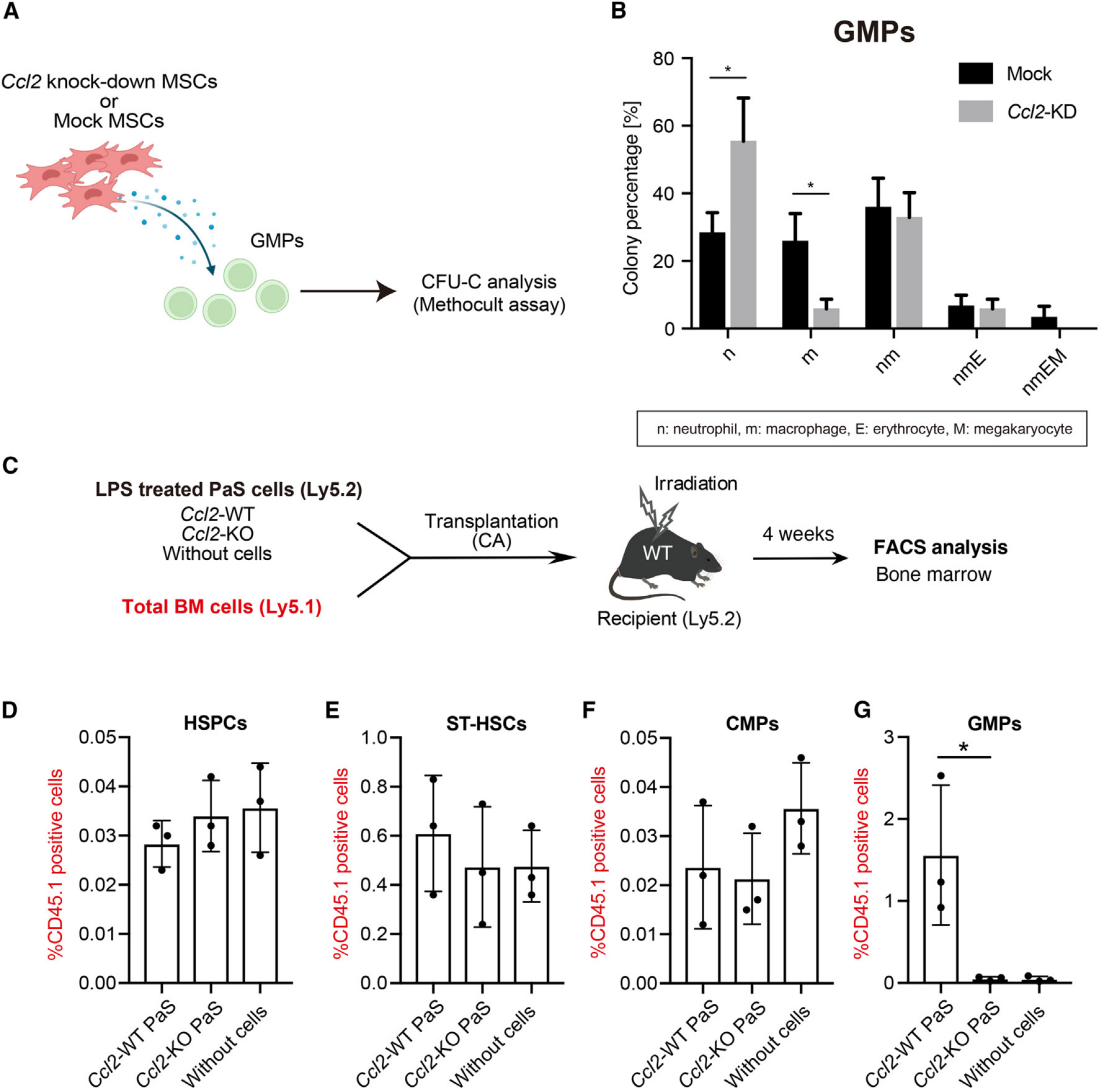
Identification of granulocyte/macrophage progenitors (GMPs) as the main source in bm response to CCL2 (A) Analysis of differentiation potential after addition of PαS-derived culture supernatant to hematopoietic progenitor cells. (B) Culture supernatants obtained from *CCL2*-KD PαS cells (CCL2-KD) and wild-type PαS cells (Mock: control vector) were added to granulocyte/macrophage progenitors (GMPs) cultured in MethoCult medium for 7 days followed by counting of colonies (E: erythrocyte, M: megakaryocyte, n: neutrophil, and m: macrophage) (n = 3). (C) Engraftment of long-term myeloid supplier via GMPs *in vivo*. LPS-treated PαS cells from *Ccr2*-KO or wild type (WT) mice (both Ly5.2) and WT BM cells (Ly5.1) were injected into irradiated WT mice (Ly5.2) via the celiac artery. (D–G) Cell subsets originating from Ly5.1 BM cells were analyzed 4 weeks after celiac artery injection. Each dot shows the percentage of cells within the Ly5.1^+^ donor-derived population (D: hematopoietic stem/progenitor cell [HSPC], E: ST-HSC, F: CMPs, and G: GMPs) (n = 3). Data are shown as mean ± standard error of mean (SEM). ^∗^p < 0.05.

To further support a specific role of CCL2 in the differentiation potential, we used a knockdown approach to suppress *Ccl2* expression in PαS cells. Conditioned media from *Ccl2* knockdown PαS cells (*Ccl2*-KD) and WT PαS cells (Mock) were incubated with GMPs (Figure 4A). Conditioned medium derived from *Ccl2*-KD cells showed a significantly lower ability to induce GMP cell differentiation into unipotent macrophages (m) than that derived from Mock cells (Figure 4B). On the other hand, the culture supernatant (*Ccl2-*KD) induced an increased rate of differentiation into unipotent neutrophils (n) when compared with the Mock. There was little change in the proportion of cell groups with multilineage differentiation (nm, nmE, and nmEM) (Figure 4B). These results indicated that PαS-secreted CCL2 played an important role in promoting the differentiation of GMPs into myeloid cells.

To examine whether transplantation of MSCs can promote myeloid differentiation *in vivo*, we performed a co-transplantation experiment with BM and PαS cells (Figure 4C). *Ccl2*-deficient PαS cells (*Ccl2*-KO PαS) or *Ccl2*-WT PαS cells (*Ccl2*-WT PαS) were transplanted into irradiated mice together with BM cells derived from Ly5.1 mice. In the group co-transplanted with *Ccl2*-WT or *Ccl2*-KO PαS cells, differentiation ratios of HSPCs, ST-HSCs, and CMPs did not vary (Figures 4D–4F); however, groups transplanted with *Ccl2*-KO PαS cells and without PαS cells showed a decrease in CD45.1-derived GMPs (Figure 4G). These data indicated that GMP engraftment into the BM or cell differentiation is promoted by PαS cell-derived CCL2.

### Alteration of human MSCs upon exogenous stimulus

**Figure 5:**
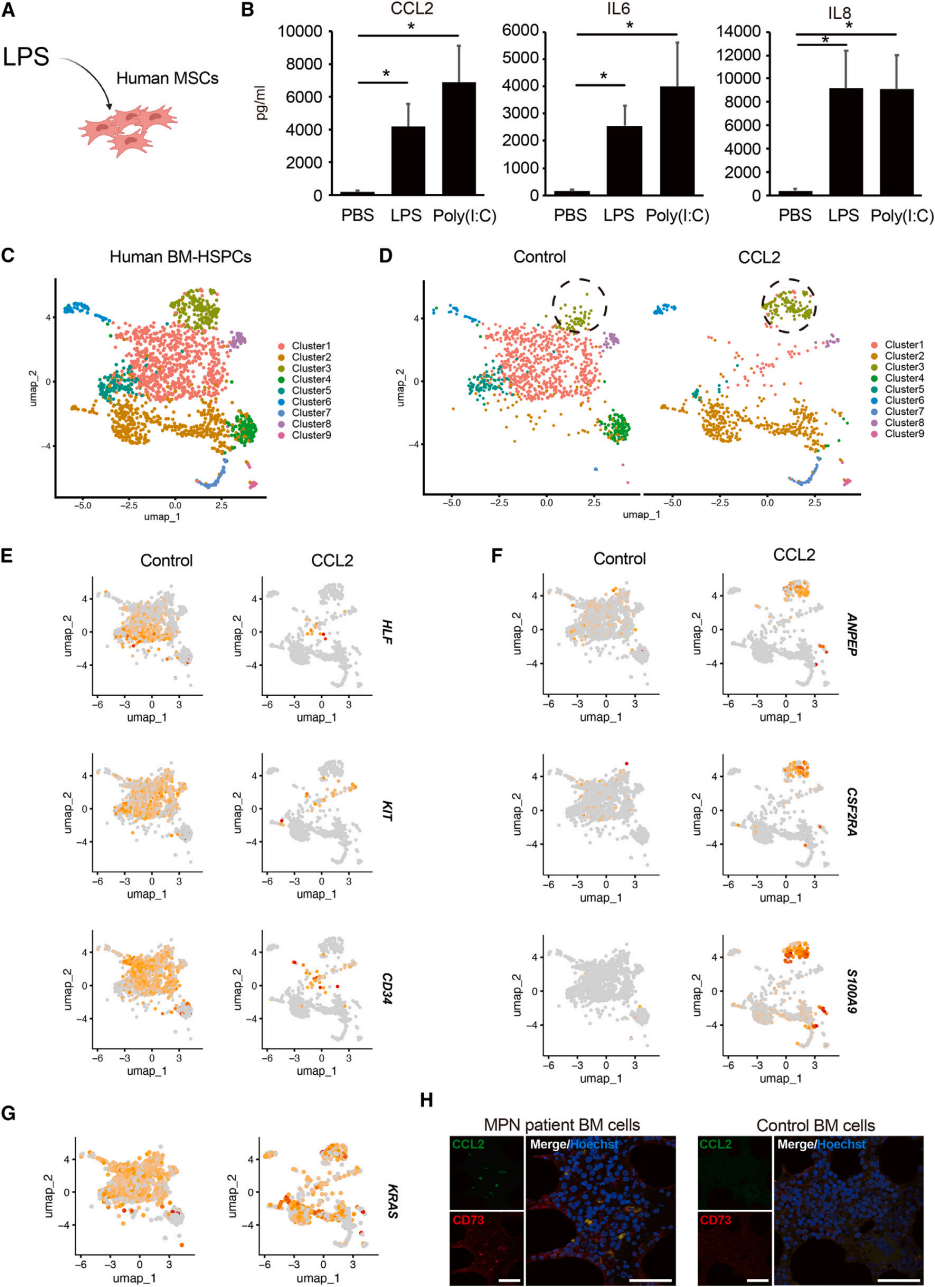
CCL2 released from normal and patient-derived MSCs (A) Analysis of human MSC culture medium after lipopolysaccharide (LPS) (500 ng/mL) and poly (I:C) (1 μg/mL) stimulation. (B) Quantification of cytokines in culture supernatants of human MSCs was performed using cytometric bead array (n = 7). Data were analyzed using Student’s t test. (C and D) UMAP plots of human BM hematopoietic stem/progenitor cells (HSPCs) using single-cell RNA sequencing (Control: without, CCL2 with stimulation). (E) Expression of HSPC markers (hepatic leukemia factor [*HLF*], mast/stem cell growth factor receptor SCFR/c-Kit [*KIT*], and *CD34*) for classification of cells. (F and G) Feature plots for expression of myeloid marker genes (*ANPEP*, *CSF2RA*, and *S100A9*), and *KRAS*. (H) Expression of CCL2 and CD73 (mesenchymal markers) in BM cells from patients with MPN. Scale bars, 50 μm. ^∗^p < 0.05. See also Figures S4 and S5.

To ascertain whether the same myeloid differentiation effect occurs in human cells, human MSCs were isolated from BM and adipose tissues, and conditioned medium was collected following LPS or Poly (I:C) stimulation (Figure 5A). The conditioned medium from CD73^+^ MSCs was confirmed to contain CCL2 as well as IL-6 and IL-8 (Figure 5B). To clarify the mechanism by which CCL2 signaling promotes the differentiation of GMPs into myeloid cells, we added CCL2 to the culture medium of human BM hematopoietic stem cells and analyzed CCL2/CCR2 downstream signals using single-cell RNA sequencing (scRNA-seq). By adding CCL2 and culturing for 10 days, we could classify the clusters representing cell populations into nine clusters (Figures 5C and 5D). In the control group (no addition of CCL2), most cells expressed hematopoietic stem cell markers (hepatic leukemia factor (*HLF*), mast/stem cell growth factor receptor SCFR/c-Kit (*KIT*), and *CD34*) (Figure 5E). On the other hand, in the clusters of the CCL2 group (with addition of CCL2), the number of undifferentiated cells decreased (Figure 5E). In addition, cluster 3, which was uniquely present after addition, expressed myeloid markers (*ANPEP*, *CSF2RA*, and *S100A9*) (Figure 5F). Furthermore, analysis of the expression of representative signaling genes downstream of CCL2 suggested that *KRAS* were expressed in myeloid cell clusters (Figure 5G). Murine BM hematopoietic stem cells and analyzed CCL2/CCR2 downstream signals using scRNA-seq show that representing cell populations into nine clusters, the major fraction (cluster 1–4; gated in dot line) was the cell population characterized by myeloid cells (Figures S4A and S4B). The expression of representative signaling genes downstream of *Ccr2* suggests that *KRas*, *Raf1*, and *Nfkb1* are expressed in myeloid cell clusters (Figure S4C). *CCL2* is overexpressed in various tumors (Nagarsheth et al., 2017). Therefore, we analyzed CCL2 expression in human MSCs (CD73-positive cells) from the BM of patients with myeloproliferative neoplasms (MPN). We found that CD73-positive cells expressed CCL2 in patients with MPN (five of six patient specimens) (Figures 5H and S5). Human MSCs were stimulated with LPS, and their conditioned medium contained CCL2. Adding CCL2 to the HSPC culture media led to fewer undifferentiated cells, with unique myeloid marker expression.

## Discussion

This study reported that MSCs and GMPs communicate in a paracrine manner via the CCL2/CCR2 signaling axis to induce early myeloid differentiation. Using complementary *in vitro* and *in vivo* models, we demonstrated that the chemokine CCL2 was secreted by MSCs (PαS cells). This study provided further evidence that the secreted CCL2, which subsequently activated the chemokine receptor, CCR2, on GMP cells directed their differentiation into CD11b-positive macrophages. The murine mechanisms described in this study occurred in a similar manner between hMSCs and hHSPCs, and these mechanisms can influence early myeloid differentiation in humans.

In a previous study, co-transplantation of mouse-derived CCR2^+^ and CCR2^-^ HSPCs into irradiated mice revealed that the CCR2^+^ group had a higher percentage of transplanted HSPC-derived CD11b-positive cells and higher expression of transcription factors PU.1 and Cebpα than the CCR2-group (Dutta et al., 2015). Both PU.1 and Cebpα are indispensable for myelopoiesis (Miyamoto et al., 2002; Reddy et al., 2002; Zhu and Emerson, 2002). Moreover, a previous study investigating the relationship between CCR2 and BM transcription factors supports our findings that CCL2 and CCR2 interaction not only assists chemotaxis, as understood for years, but also affects GMP differentiation. To obtain supportive evidence for the role of the CCL2 and CCR2 complex in the differentiation of hematopoietic cells, we transplanted total BM cells from *Ccr2*-WT and *Ccr2*-KO mice. Our results showed that the percentage of GMPs in BM and macrophages in PB significantly decreased after BM reconstitution (Figure 4G). Thus, we concluded that CCL2/CCR2 signaling significantly modulated myeloid differentiation.

Hematopoiesis is maintained by niche cells and their associated cytokines. Within the BM, MSCs interact with HSPCs and maintain them in an undifferentiated state (Mendez-Ferrer et al., 2010; Omatsu et al., 2014; Schofield, 1978). In cases of infection or inflammation, it is possible to escape from such an undifferentiated state, thus promoting the production of neutrophils and monocytes to respond to emergencies. In monocyte/macrophage differentiation, IL-1, IL-3, and/or IL-6 induce hetero-mitosis in stem cells, giving rise to new stem cells and CMPs (Valledor et al., 1998). In the presence of IL-3 and GM-CSF, M-CSF induces the proliferation of these myeloid progenitors and their differentiation into monocytic precursors (Valledor et al., 1998). In addition to normal hematopoiesis, HSPCs are activated during inflammation (Sugiyama and Nagasawa, 2012). A recent study showed that IL-7^+^ reticular mesenchymal progenitor cells controlled the number of HSPCs in the BM, and IL-7 acted as a short-range signal for CMP differentiation (Cordeiro Gomes et al., 2016). CCL2 is produced by MSCs at the site of inflammation (Shi et al., 2011). Furthermore, it can be assumed that CCL2 attracts *Ccr2*-expressing HSCs that reside in the BM cavity (Cordeiro Gomes et al., 2016; Si et al., 2010). In this study, it was possible to promote the production of CCL2 *in vivo* by transplanting activated PαS cells. Thus, our results suggest that CCL2 secretion by PαS cells might contribute to the differentiation and short-range mobilization of macrophages from GMPs.

Intravenously injected cultured MSCs do not home efficiently to the BM (Morikawa et al., 2009). Previous studies have reported that transplantation into the heart (left ventricular cavity) prevents entrapment in the lungs (Uchibori et al., 2013). When transplanted arterially (via celiac artery [CA] injection), cancer cells can be efficiently engrafted to the femur (Kuchimaru et al., 2018). Our data show that CA transplantation of cultured MSCs is useful for elucidating the effects of MSC transplantation. Cultured MSCs persist in the BM and may affect hematopoietic cells. Native CCL2 expression in PαS cells isolated from murine BM was low; however, CCL2 production was induced by LPS stimulation *in vitro* (Figure 2E). These observations agree with those of previous *in vivo* studies, wherein LPS was directly administered to mice (Shi et al., 2011). PαS cells have been found to affect HSPCs both *in vitro* and *in vivo*. In this study, we combined the following two points: (1) arterial transplantation of MSCs via the CA, and (2) transplantation of LPS-activated MSCs. These techniques enabled us to investigate the *in vivo* effects of MSC transplantation.

CCL2 levels are increased in plasma cells from the BM of patients with MPN compared with those in healthy subjects (Cominal et al., 2021). Among patients with MPN, increased CCL2 expression was observed in those with primary myelofibrosis and post-polycythemia vera/essential thrombocythemia myelofibrosis in which the CCL2 rs1024611 G/G genotype is more frequently observed than in healthy subjects (Hodeib et al., 2022). These findings in patients with MPN imply that MSCs mediate responses to stressors such as disease, aging, and infection, and that MSC-secreted factors help determine the fate of hematopoietic cells. In the future, it will be necessary to attempt to demonstrate a direct causal relationship between CCL2 and hematopoietic cells, such as analysis using large-scale patient samples, cohort analysis, and blocking analysis.

Our findings suggest a new role of CCL2 in myeloid hematopoiesis. As CCL2 secretion by MSCs significantly increased during inflammation, it can support rapid myelopoiesis. Further exploration of the role of CCL2 can lead to leveraging of CCL2- or CCL2-expressing PαS cell administration to promote rapid hematopoietic reconstitution in patients with immunosuppressed conditions following BM transplantation. This study revealed the mechanism of MSCs in directly affecting the differentiation of not only HSPCs, but also progenitor cells. Stem and progenitor cells support each other and form complex mechanisms, and elucidation of these mechanisms is important for controlling cell fate and understanding diseases.

## Experimental procedures

### Resource availability

#### Lead contact

Further information and requests for resources and reagents should be directed to and will be fulfilled by the corresponding author Chihiro Akazawa (c.akazawa.gt@juntendo.ac.jp).

#### Materials availability

This study did not generate new unique reagents.

#### Data and code availability

Raw and processed data that support the RNA-seq findings were deposited in GEO: GSE216612.

### Animal studies

We used C57BL/6J-CD45.1 (Ly5.1), C57BL/6J-CD45.1/CD45.2 (F1: Ly5.1/Ly5.2) mice (Sankyo-Laboratory Service, Tsukuba, Japan) and C57BL/6J-CD45.2 (wild type, Ly5.2) mice (Japan SLC, Shizuoka, Japan). B6.129S4-CCL2<tm1Tol/J> (*CCL2*-KO, #004434) and B6.129S4-*Ccr2*<tm1Ifc/J> (*Ccr2*-KO, #004999) mice were obtained from the Jackson Laboratory (The Jackson Laboratory, USA). Eight- to 12-week-old male mice were used as donors and recipients. All mice were housed in specific pathogen-free conditions under 12-h light/dark cycles with free access to food and water. All animal protocols were approved by the Animal Care and Use Committee of the Institute of Medical Science, University of Tokyo and Center for Experimental Animals at the Juntendo University, Japan (#1477). All animal experiments were performed in accordance with guidelines of the Laboratory Animal Experimentation at Juntendo University School of Medicine.

### Human tissue samples

Human tissue samples (MPN) were collected from individual patients at the Juntendo University. This study was approved by the Research Ethics Committee, Faculty of Medicine, Juntendo University (IRB #M16-0102, IRB #M12-0866). All patients provided written informed consent for use of their materials in this study.

### Cell culture

MSCs (PαS cells) were cultured in 10-cm dishes using Dulbecco’s modified Eagle’s medium (DMEM) supplemented with 1% HEPES (Gibco, Waltham, MA, USA), 1% penicillin/streptomycin (Gibco), 20% fetal bovine serum (FBS), and basic fibroblast growth factor (5 ng/mL; Pepro Tech, Cranbury, NJ, USA). The medium was replaced every 3–4 days. Before MSCs became confluent, they were passaged and cryopreserved. MSCs were cultured and used for further experiments at passage 3–4. For co-culturing HSPCs with MSCs, 1.0 × 10^4^ PαS cells/24-wells were seeded 2 days before seeding 1.0 × 10^4^ HSPCs on a cell culture insert (Corning, Durham, NC, USA, 0.4 μm pore size). For collecting conditioned medium, the culture medium was replaced with S-clone (Iwai North America, San Carlos, CA), and cells were further cultured for another 24 h before collecting the conditioned medium. The MSC conditioned medium was collected and centrifuged at 300 *× g* for 3 min to remove debris. HSPCs were cultured with MSC conditioned medium or control medium (S-clone) for 6 days. For analysis of the differentiation ability of HSPC-derived cells, the percentages of myeloid lineage (CD11b^+^/Gr-1^+^) cells, lymphoid lineage (CD3e^+^/CD45R^+^) cells, and erythroblasts (Ter119^+^ cells) were measured via flow cytometry (FACSAria II, BD Biosciences).

### Cell isolation using flow cytometry

For CD45^−^CD31^−^Ter119^−^PDGFRα^+^Sca-1^+^ cell (MSCs: PαS cells) preparation, the femurs, tibias, and ilea were dissected and crushed using a pestle. Bone fragments were collected and incubated for 1 h at 37°C in DMEM (Wako, Osaka, Japan) containing 0.2% collagenase (Wako), 10 mM HEPES (FUJIFILM Wako Pure Chemical, Osaka, Japan), and 1% penicillin/streptomycin. After filtration using a 70-μm cell strainer to remove debris and bone fragments, cells were stained with APC-conjugated mouse PDGFRα (clone APA5), FITC-conjugated mouse Sca-1 (clone D7), PE-cy7-conjugated mouse CD45 (clone 30-F11), mouse TER119 (clone TER-119), and mouse CD31 (clone 390) antibodies (BD Biosciences, Franklin Lakes, NJ, USA). For preparation of c-Kit^+^Sca-1^+^Lineage^−^ (HSPCs: KSL cells) and progenitor cells, mouse BM cells were stained with eFluor450-conjugated mouse CD34 (clone: RAM34, eBioscienes, San Diego, CA, USA), PE-conjugated Sca-1 (clone D7), APC-conjugated c-Kit (clone 2B8), PE-cy7-conjugated CD3e (clone 145-2C11), CD45R (clone RA3-6B2), Ter119 (clone TER119), Gr-1 (clone RB6-8C5), CD11b (clone M1/70), and BV786-conjugated CD16/32 (FcγR) (clone 2.4G2) antibodies (BD Biosciences). Cell sorting and data acquisition were performed using FACSAria II or FACSVerse (BD Biosciences), and data were analyzed using the FlowJo (v10.5.3) software (BD Biosciences).

### LPS administration

PαS cells (5.0 × 10^5^ cells/6 wells) were cultured, and LPS (Sigma-Aldrich, Saint Louis, MO, USA) was added to the medium (100 ng/mL). After 4 h of LPS addition, the culture medium was changed to S-clone (Iwai North America), and cells were cultured for 24 h (LPS-treated MSC conditioned medium). HSPCs were cultured in S-clone (SCF 50 ng/mL and TPO 50 ng/mL)/LPS-treated MSC conditioned medium (1:1) for 6 days. S-clone was added to the control group instead of the condition medium. Percentages of myeloid lineage (CD11b^+^/Gr-1^+^) cells were measured via flow cytometry. The levels of cytokines were measured using the Cytometric Beads Array (BD Biosciences), and analysis was performed using FACSVerse (BD Biosciences). For *in vivo* LPS administration, LPS was intraperitoneally administered to adult mice (35 μg). After 4 h, the femur and tibia were collected, cell populations (PaS, non-PaS, CD31-positive endothelial cells, lineage^+^ mature hematopoietic cells) were isolated using a flow cytometer, and RNA was collected. Expression analysis of the *CCL2* gene in each cell population was performed using quantitative PCR. The ratios of CMPs (c-Kit^+^Sca-1^−^Lineage^−^FcγR^−^ cells), GMPs (c-Kit^+^Sca-1^−^Lineage^−^FcγR^+^ cells), and myeloid cells were analyzed by sorting using a FACSAria II (BD Biosciences).

### BM transplantation assay

A total of 1 × 10^6^ BM cells (*Ccr*2-WT or *Ccr2*-KO cells from male C57BL/6-CD45.2) were transplanted via single retro-orbital injection into irradiated (9.5 Gy) recipient mice (C57BL/6-CD45.1) along with 1 × 10^6^ whole BM competitor cells (male C57BL/6-CD45.1/CD45.2). PB analysis was performed every 4 weeks. The percentage of cells expressing Ly5.2 among donor-derived cells (*Ccr*2-KO or *Ccr*2-WT cells) was analyzed using a flow cytometer (FACSAria II, BD Biosciences). BM and PB cells were stained using an FITC-conjugated Ly5.2 (clone: eBioscienes), and data were analyzed using the FlowJo (v10.5.3) software (BD Biosciences).

### Co-transplantation of HSPCs and MSCs

PαS cells were treated with 100 ng/mL of LPS (L2654, Sigma-Aldrich, Saint Louis, MO, USA) for 4 h and harvested. Following this, 1 × 10^5^ LPS-treated PαS cells (CCL2-WT or CCL2-KO cells from male C57BL/6-CD45.2) were transplanted via caudal artery injection into irradiated (9.5 Gy) recipient mice (C57BL/6-CD45.2) along with 1 × 10^6^ whole BM cells (male C57BL/6-CD45.1). Whole BM cell-derived donor cells (Ly5.1) were analyzed after 4 weeks of transplantation. The percentage of HSPCs, ST-HSCs, CMPs, and GMPs expressing Ly5.1 among donor-derived cells (whole BM cells) was analyzed using a flow cytometer (FACSAria II, BD Biosciences).

### Human MSC isolation

Human adipose tissue was treated with collagenase to dissociate it into single cells, and it was stained with APC-conjugated anti-CD73 (BioLegend, San Diego, CA, USA). To distinguish between living and dead cells, cells were suspended in propidium iodide solution, and this was followed by sorting using a FACSAria II (BD Biosciences). All experiments were analyzed using FlowJo software ver.10.8.1 (BD Biosciences). CD73^+^ cells were isolated from human adipose tissues as previously described (Suto et al., 2017, 2020).

Human adipose-derived MSCs (CD73^+^ cells) were cultured in DMEM-Gluta MAX (Gibco) containing 20% FBS, 1% penicillin/streptomycin, and 20 ng/mL bFGF (REPROCELL, Kanagawa, Japan) as the MSC medium. Cells were grown to 70%–80% confluency in the MSC medium; following this, fresh medium containing LPS (500 ng/mL) or poly (I:C) (1 μg/mL) was added, and the cells were incubated for 1 h. Cells were washed twice in MSC medium. After 24 h, the conditioned media was collected. Cytokine levels were measured using the Cytometric Beads Array (BD Biosciences).

### Single-cell RNA sequencing analysis

Mouse HSPCs and human BM CD34^+^ cells were cultured at 37°C under 5% CO_2_ with CCL2 (Pepro tech) for 10 days (with CCL2 or PBS). From each cell culture, a propidium iodide-negative fraction was sorted using MoFlo (Beckman Coulter), and single-cell gel beads-in-emulsions were generated using the Chromium Controller (10× Genomics, Pleasanton, CA, USA). Libraries were generated using the Single Cell 3′ Reagent Kit version 3.1 (10× Genomics) according to the manufacturer’s instructions. Cells were sequenced on Illumina Hiseq X (Macrogen Inc, Seoul, South Korea). Sequence data were aligned by the reference genome (GRCh38) using the Cellranger v6.1.1 pipeline. Subsequent analysis was performed using Seurat v4.047 in R. Using Read10X function, we read the datasets and returned the unique molecular identified count matrix of each data.

### Immunohistochemistry

Formalin-fixed paraffin-embedded BM specimens were mounted on slides, and then stained with CCL2 (mouse anti-CCL2, R&D systems, MAB679-100) and CD73 antibody (rabbit anti-CD73, abcam, ab175396, 1:100). The primary antibodies were incubated with 2% BSA/PBS at 4°C overnight. The cells were washed with PBS and stained with anti-rabbit Alexa Fluor 594 (A11037, 1:1000), goat anti-mouse IgG2b Alexa Fluor 488 (A21141, 1:1000) (Life Technologies, Austin, TX, USA), and Hoechst 33342 (#PN226, 1:500, Dojindo, Kumamoto, Japan).

### Statistical analysis

Quantitative data are presented as mean ± standard error of the mean (SEM) of at least three representative experiments. Statistical analyses of the gene expression were performed using one-way ANOVA with a Bonferroni post hoc analysis for comparison of three or more groups. For comparisons between groups, Student’s t test was used. ^∗^p < 0.05 and ^∗∗^p < 0.01 were considered significant. GraphPad Prism 7 ver. 7.0d (GraphPad Software, San Diego, CA, USA) was used for statistical analysis.

## Supplemental information

Document S1. Figures S1–S5

Document S2. Article plus supplemental information

## References

[bib1] Bianco P., Robey P.G., Simmons P.J. (2008). Mesenchymal stem cells: revisiting history, concepts, and assays. Cell Stem Cell.

[bib2] Cominal J.G., Cacemiro M.D.C., Berzoti-Coelho M.G., Pereira I.E.G., Frantz F.G., Souto E.X., Covas D.T., de Figueiredo-Pontes L.L., Oliveira M.C., Malmegrim K.C.R., de Castro F.A. (2021). Bone Marrow Soluble Mediator Signatures of Patients With Philadelphia Chromosome-Negative Myeloproliferative Neoplasms. Front. Oncol..

[bib3] Cordeiro Gomes A., Hara T., Lim V.Y., Herndler-Brandstetter D., Nevius E., Sugiyama T., Tani-Ichi S., Schlenner S., Richie E., Rodewald H.R. (2016). Hematopoietic Stem Cell Niches Produce Lineage-Instructive Signals to Control Multipotent Progenitor Differentiation. Immunity.

[bib4] da Silva C.L., Gonçalves R., Crapnell K.B., Cabral J.M.S., Zanjani E.D., Almeida-Porada G. (2005). A human stromal-based serum-free culture system supports the ex vivo expansion/maintenance of bone marrow and cord blood hematopoietic stem/progenitor cells. Exp. Hematol..

[bib5] Dutta P., Sager H.B., Stengel K.R., Naxerova K., Courties G., Saez B., Silberstein L., Heidt T., Sebas M., Sun Y. (2015). Myocardial Infarction Activates CCR2(+) Hematopoietic Stem and Progenitor Cells. Cell Stem Cell.

[bib6] Hodeib H., Abd El Hai D., Tawfik M.A., Allam A.A., Selim A., Elsawy A.A., Youssef A. (2022). CCL2 rs1024611Gene Polymorphism in Philadelphia-Negative Myeloproliferative Neoplasms. Genes.

[bib7] Iwasaki H., Akashi K. (2007). Myeloid lineage commitment from the hematopoietic stem cell. Immunity.

[bib8] Kuchimaru T., Kataoka N., Nakagawa K., Isozaki T., Miyabara H., Minegishi M., Kadonosono T., Kizaka-Kondoh S. (2018). A reliable murine model of bone metastasis by injecting cancer cells through caudal arteries. Nat. Commun..

[bib35] Mabuchi Y., Morikawa S., Harada S., Niibe K., Suzuki S., Renault-Mihara F., Houlihan D.D., Akazawa C., Okano H., Matsuzaki Y. (2013). LNGFR(+)THY-1(+)VCAM-1(hi+) cells reveal functionally distinct subpopulations in mesenchymal stem cells. Stem Cell Reports.

[bib9] Mabuchi Y., Matsuzaki Y. (2016). Prospective isolation of resident adult human mesenchymal stem cell population from multiple organs. Int. J. Hematol..

[bib10] Mehta H.M., Malandra M., Corey S.J. (2015). G-CSF and GM-CSF in Neutropenia. J. Immunol..

[bib11] Méndez-Ferrer S., Michurina T.V., Ferraro F., Mazloom A.R., Macarthur B.D., Lira S.A., Scadden D.T., Ma'ayan A., Enikolopov G.N., Frenette P.S. (2010). Mesenchymal and haematopoietic stem cells form a unique bone marrow niche. Nature.

[bib12] Miyamoto T., Iwasaki H., Reizis B., Ye M., Graf T., Weissman I.L., Akashi K. (2002). Myeloid or lymphoid promiscuity as a critical step in hematopoietic lineage commitment. Dev. Cell.

[bib13] Morikawa S., Mabuchi Y., Kubota Y., Nagai Y., Niibe K., Hiratsu E., Suzuki S., Miyauchi-Hara C., Nagoshi N., Sunabori T. (2009). Prospective identification, isolation, and systemic transplantation of multipotent mesenchymal stem cells in murine bone marrow. J. Exp. Med..

[bib14] Nagarsheth N., Wicha M.S., Zou W. (2017). Chemokines in the cancer microenvironment and their relevance in cancer immunotherapy. Nat. Rev. Immunol..

[bib15] Omatsu Y., Seike M., Sugiyama T., Kume T., Nagasawa T. (2014). Foxc1 is a critical regulator of haematopoietic stem/progenitor cell niche formation. Nature.

[bib16] Osawa M., Hanada K., Hamada H., Nakauchi H. (1996). Long-term lymphohematopoietic reconstitution by a single CD34-low/negative hematopoietic stem cell. Science.

[bib17] Pittenger M.F., Mackay A.M., Beck S.C., Jaiswal R.K., Douglas R., Mosca J.D., Moorman M.A., Simonetti D.W., Craig S., Marshak D.R. (1999). Multilineage potential of adult human mesenchymal stem cells. Science.

[bib18] Reddy V.A., Iwama A., Iotzova G., Schulz M., Elsasser A., Vangala R.K., Tenen D.G., Hiddemann W., Behre G. (2002). Granulocyte inducer C/EBPalpha inactivates the myeloid master regulator PU.1: possible role in lineage commitment decisions. Blood.

[bib19] Schofield R. (1978). The relationship between the spleen colony-forming cell and the haemopoietic stem cell. Blood Cell.

[bib20] Seita J., Sahoo D., Rossi D.J., Bhattacharya D., Serwold T., Inlay M.A., Ehrlich L.I.R., Fathman J.W., Dill D.L., Weissman I.L. (2012). Gene Expression Commons: an open platform for absolute gene expression profiling. PLoS One.

[bib21] Serbina N.V., Pamer E.G. (2006). Monocyte emigration from bone marrow during bacterial infection requires signals mediated by chemokine receptor CCR2. Nat. Immunol..

[bib22] Shi C., Jia T., Mendez-Ferrer S., Hohl T.M., Serbina N.V., Lipuma L., Leiner I., Li M.O., Frenette P.S., Pamer E.G. (2011). Bone marrow mesenchymal stem and progenitor cells induce monocyte emigration in response to circulating toll-like receptor ligands. Immunity.

[bib23] Si Y., Tsou C.L., Croft K., Charo I.F. (2010). CCR2 mediates hematopoietic stem and progenitor cell trafficking to sites of inflammation in mice. J. Clin. Invest..

[bib24] Sugiyama T., Kohara H., Noda M., Nagasawa T. (2006). Maintenance of the hematopoietic stem cell pool by CXCL12-CXCR4 chemokine signaling in bone marrow stromal cell niches. Immunity.

[bib25] Sugiyama T., Nagasawa T. (2012). Bone marrow niches for hematopoietic stem cells and immune cells. Inflamm. Allergy - Drug Targets.

[bib26] Suto E.G., Mabuchi Y., Suzuki N., Suzuki K., Ogata Y., Taguchi M., Muneta T., Sekiya I., Akazawa C. (2017). Prospectively isolated mesenchymal stem/stromal cells are enriched in the CD73(+) population and exhibit efficacy after transplantation. Sci. Rep..

[bib27] Suto E.G., Mabuchi Y., Toyota S., Taguchi M., Naraoka Y., Itakura N., Matsuoka Y., Fujii Y., Miyasaka N., Akazawa C. (2020). Advantage of fat-derived CD73 positive cells from multiple human tissues, prospective isolated mesenchymal stromal cells. Sci. Rep..

[bib28] Teleb R.S., Abdul-Hafez A., Othman A., Ahmed A.E., Elsaid A.A., Arif H., Zarea A.A., Abdulmageed M., Mohamed H., Ibrahim S.A. (2023). Cord Blood Plasma and Placental Mesenchymal Stem Cells-Derived Exosomes Increase Ex Vivo Expansion of Human Cord Blood Hematopoietic Stem Cells While Maintaining Their Stemness. Cells.

[bib29] Uchibori R., Tsukahara T., Mizuguchi H., Saga Y., Urabe M., Mizukami H., Kume A., Ozawa K. (2013). NF-kappaB activity regulates mesenchymal stem cell accumulation at tumor sites. Cancer Res..

[bib30] Valledor A.F., Borràs F.E., Cullell-Young M., Celada A. (1998). Transcription factors that regulate monocyte/macrophage differentiation. J. Leukoc. Biol..

[bib31] Wilkinson A.C., Ishida R., Kikuchi M., Sudo K., Morita M., Crisostomo R.V., Yamamoto R., Loh K.M., Nakamura Y., Watanabe M. (2019). Long-term ex vivo haematopoietic-stem-cell expansion allows nonconditioned transplantation. Nature.

[bib32] Zhou B.O., Yue R., Murphy M.M., Peyer J.G., Morrison S.J. (2014). Leptin-receptor-expressing mesenchymal stromal cells represent the main source of bone formed by adult bone marrow. Cell Stem Cell.

[bib33] Zhu J., Emerson S.G. (2002). Hematopoietic cytokines, transcription factors and lineage commitment. Oncogene.

[bib34] Zimmerli W., Zarth A., Gratwohl A., Speck B. (1991). Neutrophil function and pyogenic infections in bone marrow transplant recipients. Blood.

