# Supplementary material for: Activated mesenchymal stem/stromal cells promote myeloid cell differentiation via CCL2/CCR2 signaling
Source: Stem Cell Reports. 2024 Feb 29;19(3):414–25. doi: 10.1016/j.stemcr.2024.02.002 (PMC10937152; doi:10.1016/j.stemcr.2024.02.002)
Supplement: Document S1. Figures S1–S5 [file mmc1.pdf]

Stem Cell Reports, Volume *19*

Supplemental Information

Activated mesenchymal stem/stromal cells promote myeloid cell differentiation via CCL2/CCR2 signaling

Satoshi Yamazaki, Yo Mabuchi, Takaharu Kimura, Eriko Grace Suto, Daisuke Hisamatsu, Yuna Naraoka, Ayako Kondo, Yuzuki Azuma, Riko Kikuchi, Hidekazu Nishikii, Soji Morishita, Marito Araki, Norio Komatsu, and Chihiro Akazawa

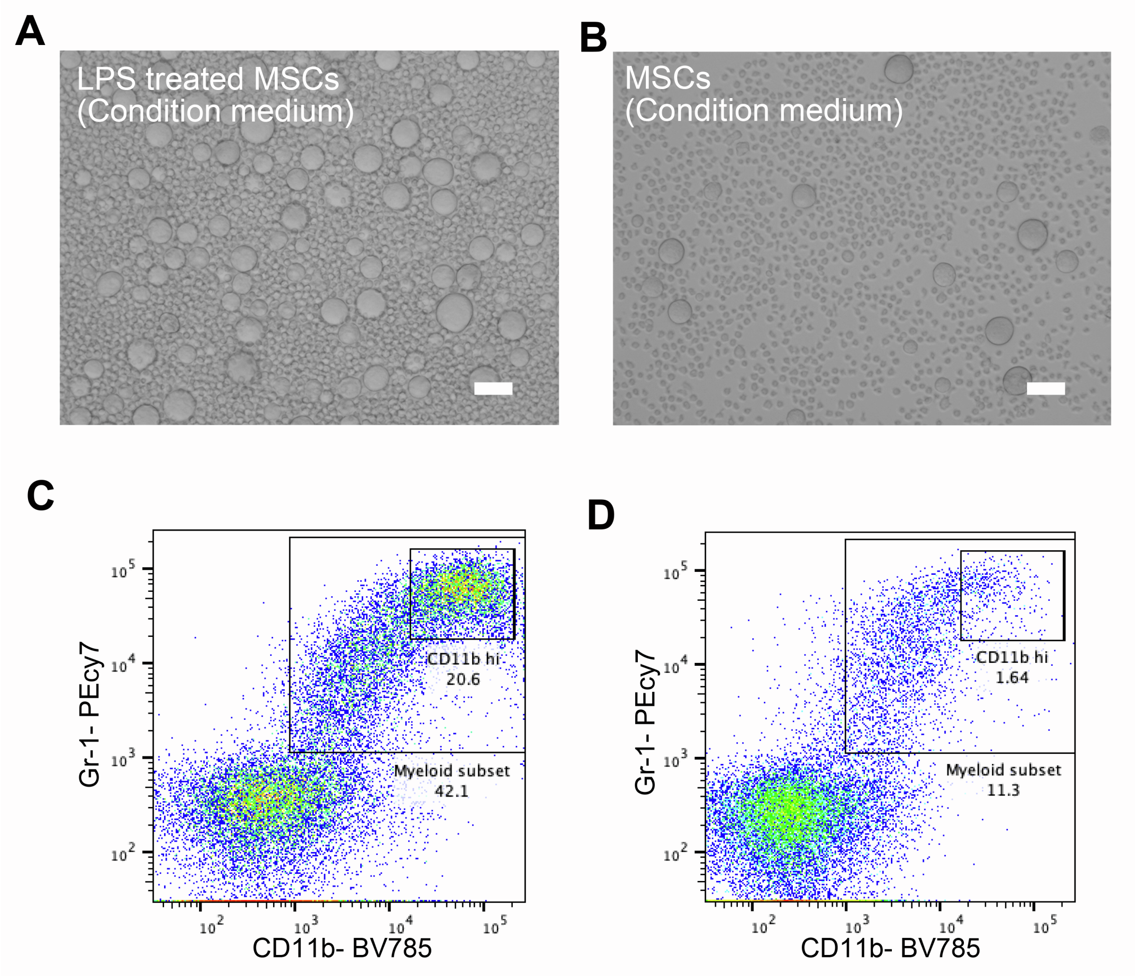

Supplementary Fig. S1: Analysis of cell morphology and cell surface antigens of hematopoietic stem/progenitor cell (HSPC)-derived cells after addition of condition medium. The cell morphology and cell surface antigens in mesenchymal stem cell (MSC) condition medium after lipopolysaccharide (LPS) stimulation. **(A, B)** Phase image of HSPCs after addition of condition medium (a, LPS treated MSC condition medium, b, MSC condition medium). **(C, D)** Representative FACS profiles show the proportion of myeloid lineage (CD11b+/Gr-1+) cells (C, LPS treated MSC condition medium, D, MSC condition medium). Scale bars, 50 um.

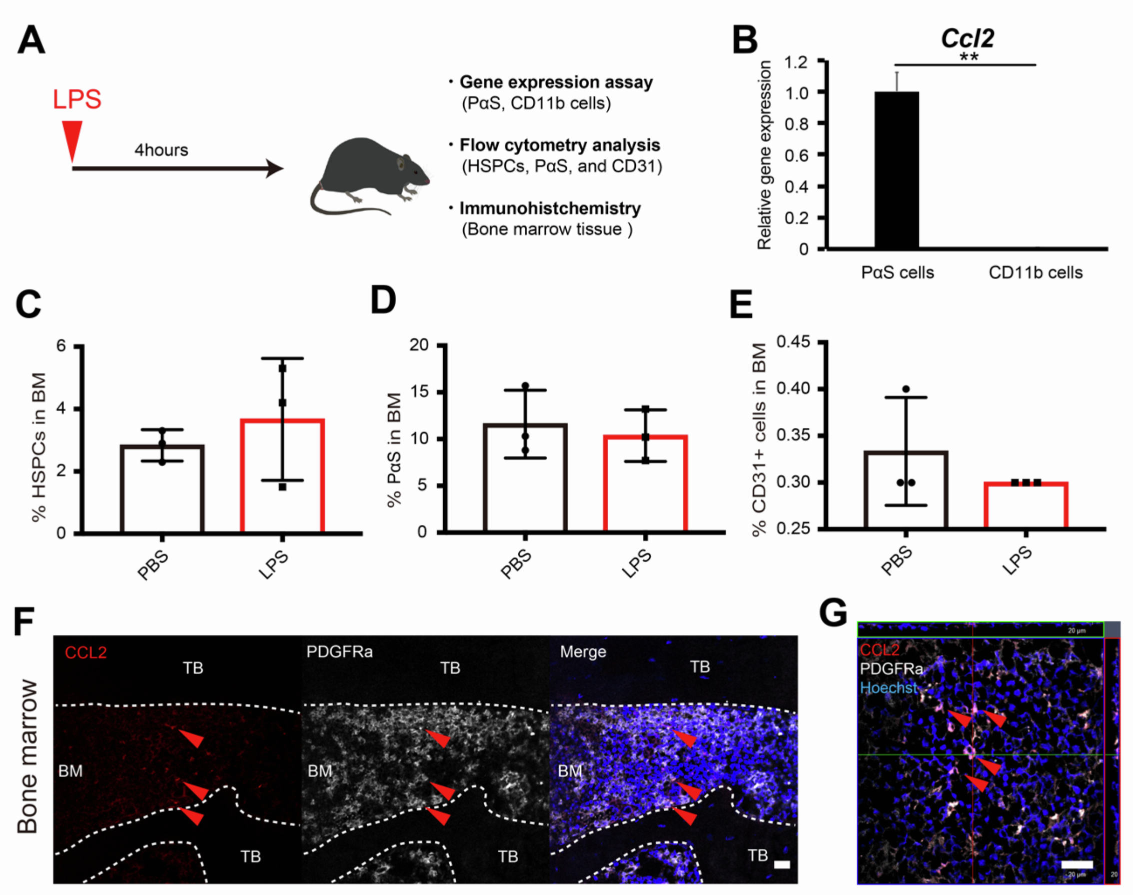

Supplementary Fig. S2: Effects of inflammation on blood and mesenchymal stem cells (MSCs) in an acute inflammation model. (A) Experimental scheme of the analysis of the effect of LPS stimulation on bone marrow (BM) **(B)** *Ccl2* gene expression levels in BM cells (PαS cells, and CD11b+ cells). **(C-E)** Bar graph shows cell ratio *in vivo* after LPS or phosphate-buffered saline (PBS) administration (HSPCs, PaS, and CD31 cells) (n=3). **(F, G)** Immunohistochemical analysis of mouse BM after LPS administration. Ccl2- (red) and PDGFRα-positive cells (white), Hoechst (blue) in BM. Red arrow head indicates Ccl2 and PDGFRα double-positive cells. Scale bars, 20 um.

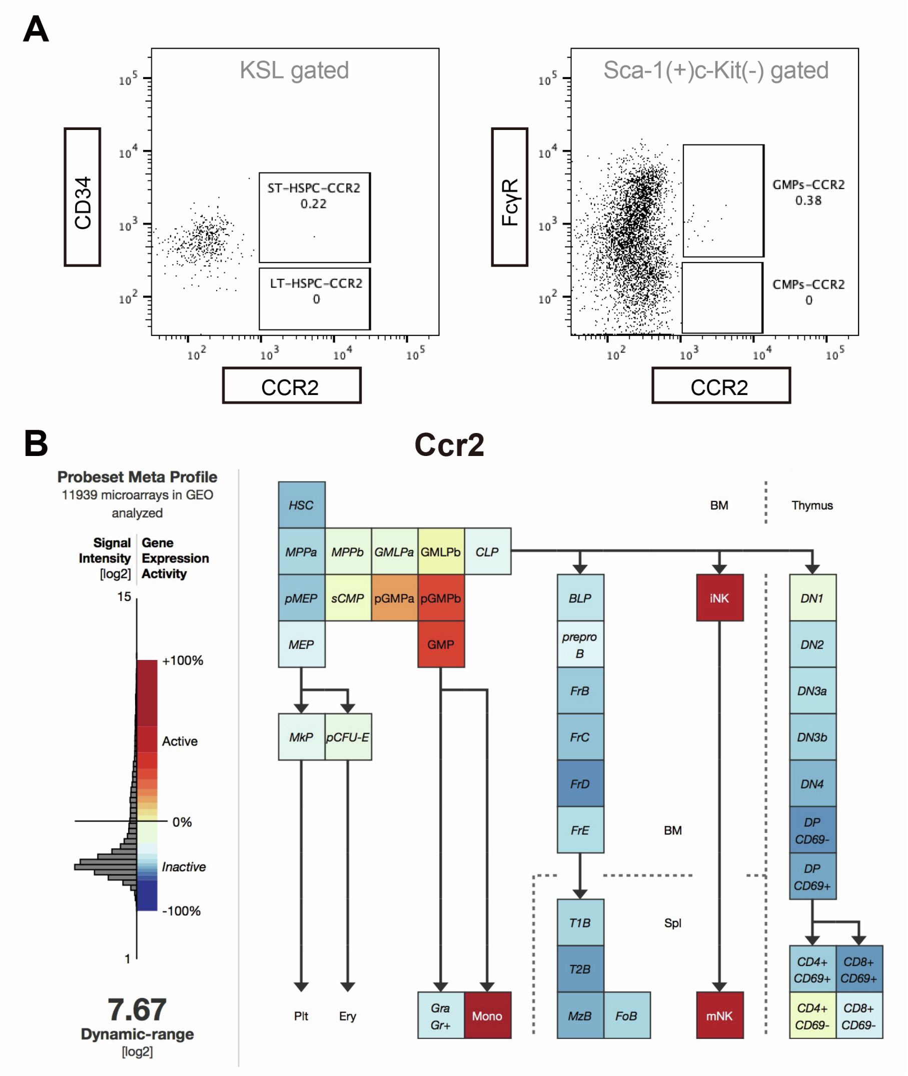

Supplementary Fig. S3: Expression analysis of Ccr2 gene in blood cell lineage.

(A) Expression analysis of Ccr2 receptor in hematopoietic cells. The expression of Ccr2 present in mouse BM was analyzed using a flow cytometer. **(B)** We investigated the expression of CCR2 in blood cell types using a microarray-based database by Gene Expression Commons (https://gexc.stanford.edu/)

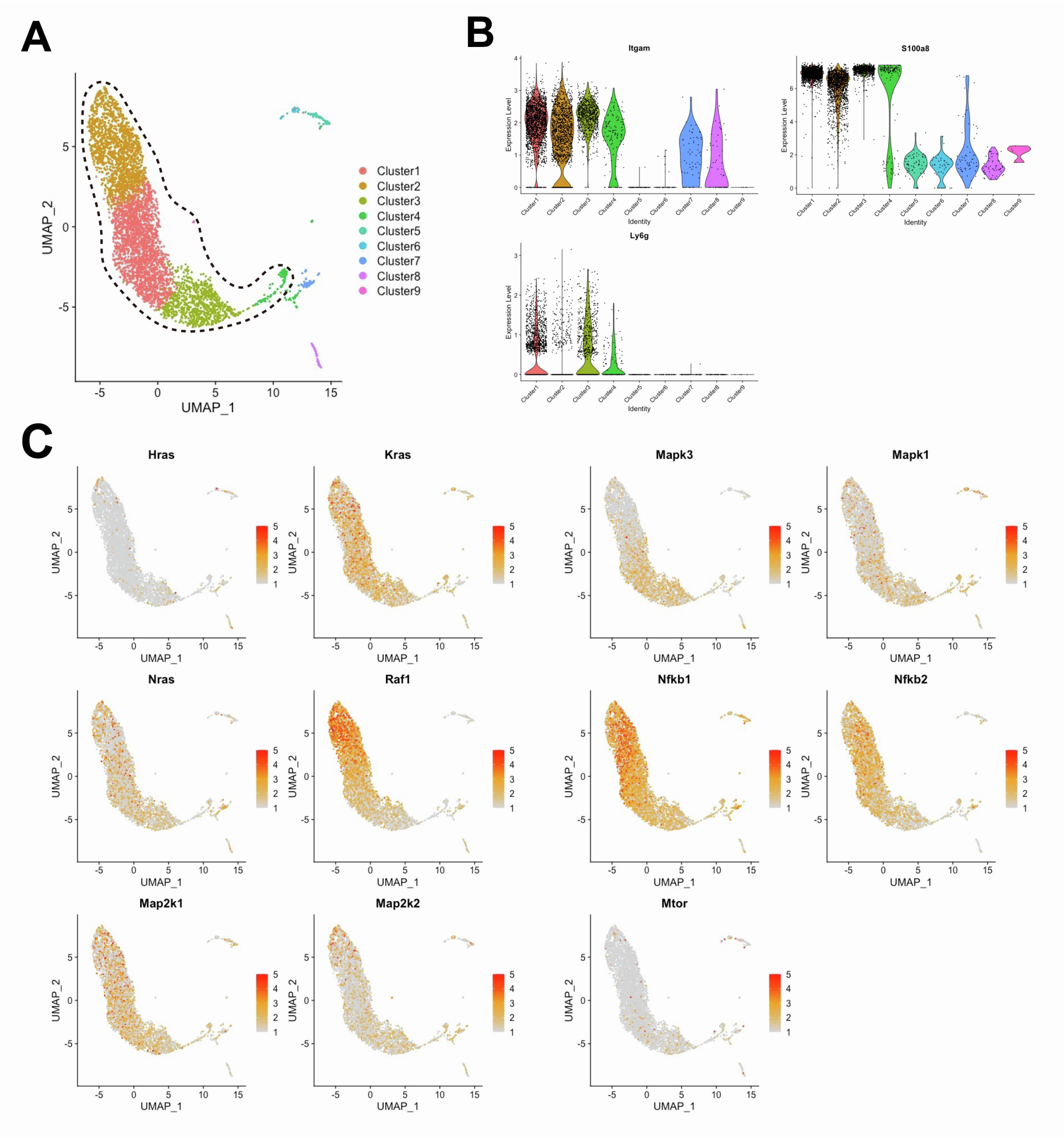

Supplementary Fig. S4: Analysis of mouse bone marrow derived HSPCs after Ccl2 stimulation. (A) UMAP plots of mouse HSPC after stimulating Ccl2. **(B)** Expression of myeloid markers (*Itgam*, *Ly6g*, *and S100a8*) classification of cells in mouse HSPCs. **(C)** Feature plots for the expression of selected marker genes: *Hras, Kras, Mapk3, Mapk1, Nras, Raf1, Nfkb1, Nfkb2, Map2k1, Map2k2,* and *Mtor*.

No,	Tissue	Diseases	CCL2 expression in CD73 cells	Percentage
No,1	BM	MPN (PV, VF)	Positive	66%
No,2	BM	MPN (PV, VF)	Positive	46%
No,3	BM	MPN (ET, VF)	Positive	87%
No,4	BM	MPN (ET, VF)	Positive	81%
No,5	BM	MPN (ET, Del52)	Negative	-
No,6	BM	MPN (ET, Del52)	Positive	30%
No,7	BM	Control	Positive	90%
No,8	BM	Control	Positive	88%
No,9	BM	Control	Negative	-
No,10	BM	Control	Negative	-

Supplementary Fig. S5: Patient specimen information.

BM: bone marrow, MPN: myeloproliferative neoplasms, PV: polycythemia vera, ET: Essential thrombocythemia, CCL2 expression in CD73 cells: Percentage of CD73 and CCL2 positive cells in bone marrow.
